# Detection of alpha particle emitters originating from nuclear fuel inside reactor building of Fukushima Daiichi Nuclear Power Plant

**DOI:** 10.1038/s41598-018-36962-4

**Published:** 2019-01-24

**Authors:** Yuki Morishita, Tatsuo Torii, Hiroshi Usami, Hiroyuki Kikuchi, Wataru Utsugi, Shiro Takahira

**Affiliations:** 1 Collaborative Laboratories for Advanced Decommissioning Science (CLADS), Japan Atomic Energy Agency, 790-1 Motooka Ohtsuka, Tomioka Town, Futaba-gun, Fukushima 979-1151 Japan; 2 Tokyo Electric Power Company Holdings, Inc., 22 Kitahara, Ottozawa, Ohkuma-machi, Futaba-gun, Fukushima 979-1301 Japan

## Abstract

We measured alpha emitters obtained from a reactor building in the Fukushima Daiichi Nuclear Power Plant (FDNPP) by using an alpha particle imaging detector. For developing the detector, we used a very thin (0.05-mm-thick) a cerium-doped Gd_3_(Ga,Al)_5_O_12_ (Ce:GAGG) scintillator and silicon photomultiplier (SiPM) arrays as the photodetector. The floor of the reactor building in FDNPP was wiped off by using smear papers, and the radioactivity of these papers was measured by the alpha particle imaging detector. In addition, we measured a Plutonium (Pu) sample (mainly 5.5 MeV alpha particles from ^238^Pu) obtained from a nuclear fuel facility by using of the same detector for comparison with the smear papers. The alpha spectrum was in the energy range of 5–6 MeV, which corresponds to the alpha particle energy of ^238^Pu (5.5 MeV). The correlation coefficient of the alpha spectra of the smear papers and the Pu sample had a strong positive linear relation. Moreover, the peak of ^241^Am was identified by gamma spectrum measurement. Based on these results, we report actual findings of alpha emitters in the FDNPP reactor buildings originating from nuclear fuels. The surface contamination level of alpha emitters exceeded 4 Bq/cm^2^.

## Introduction

After the Great East Japan Earthquake hit the Fukushima Daiichi Nuclear Power Plant (FDNPP), large quantities of radioactive materials were released inside and outside of the FDNPP^1–6^. Especially, a reactor building of the FDNPP was seriously contaminated by radioactive materials released from the Primary Containment Vessel. These radioactive materials were mostly beta and gamma emitters such as ^137^Cs and ^90^Sr, but there is a high possibility that alpha emitters such as ^238^Pu and ^239^Pu were released as well^7,8^. As the decommissioning process continues, the frequency with which workers enter highly contaminated areas such as the reactor building will surely increase. Information about the activity and radionuclides of alpha emitters at a working site is extremely important for preventing workers from exposure to radiation^9–11^. For instance, based on radionuclide information, a person working in the reactor building of the FDNPP can choose suitable protection gear (e.g., protection mask). Identification of the radionuclides of the alpha emitters is important because conversion factor to internal exposure changes largely based on the radionuclides of the alpha emitters^12^. However, measuring alpha activity directly at the FDNPP site is extremely difficult because the dose-rate of gamma rays there is higher than 10 mSv/h^13^, which makes it impossible for workers to stay in the field for any significant duration. Therefore, alpha emitters released from the FDNPP has been measured only in environments outside of the FDNPP site^7,8,14–16^, and very few measurements have been conducted of alpha emitters inside the FDNPP site.

Using silver-doped zinc sulfide (ZnS(Ag)) survey meters used to be the only option for measuring alpha emitters at the FDNPP site. Such survey meters have been used for measuring alpha particle count rates. However, the energy resolutions obtained by the ZnS(Ag) scintillators are rather low, therefore, not showing where the alpha emitters are originating from: nuclear fuels or naturally occurring radionuclide such as Radon (Rn) and progeny^17–21^. To identify a radionuclide of an alpha emitter, an alpha particle detector with high energy resolution is required^22^.

Another aim of measuring alpha emitters is obtaining information about the two-dimensional distribution of alpha emitters. In the previous study, alpha particles from Pu characteristically show up as spots in the two-dimensional distributions^20^. Information about the size and distribution of alpha emitters is very useful for radiation protection. For example, the particle size of alpha emitters is important for estimating internal exposure to a worker^12^. Moreover, information about the two-dimensional distribution of alpha emitters is valuable for distinguishing Pu from Rn progeny because the distributions of alpha particles between them are different^20,23,24^. An imaging plate (IP) has been widely used to obtain the two-dimensional distribution of alpha emitters^25,26^, but this plate is sensitive to beta particles and gamma rays as well. There are large quantities of beta and gamma emitters at the FDNPP site. The IP cannot distinguish alpha particles from beta particles and gamma rays and identify radionuclides of alpha emitters. Therefore, energy information is necessary to measure only alpha emitters in FDNPP.

We have developed an alpha particle imaging detector that can measure the energy spectrum and the two-dimensional distribution of alpha particles by combining a cerium-doped Gd_3_(Ga,Al)_5_O_12_ (Ce: GAGG) scintillator with a silicon photomultiplier (SiPM). This detector has good energy resolution (~13% full width at half maximum (FWHM)) and high spatial resolution (0.6 mm FWHM) for 5.5 MeV alpha particles^27^. Furthermore, its beta and gamma sensitivity can be reduced by setting the thickness of the Ce: GAGG scintillator to 0.05 mm.

In this study, we measured alpha emitters in the reactor building of FDNPP for the first time by using the developed alpha particle imaging detector. The features of radionuclides of alpha emitters in the reactor building of FDNPP were clarified by these measurements.

## Results

### Preparation of smear papers

**Figure 1:**
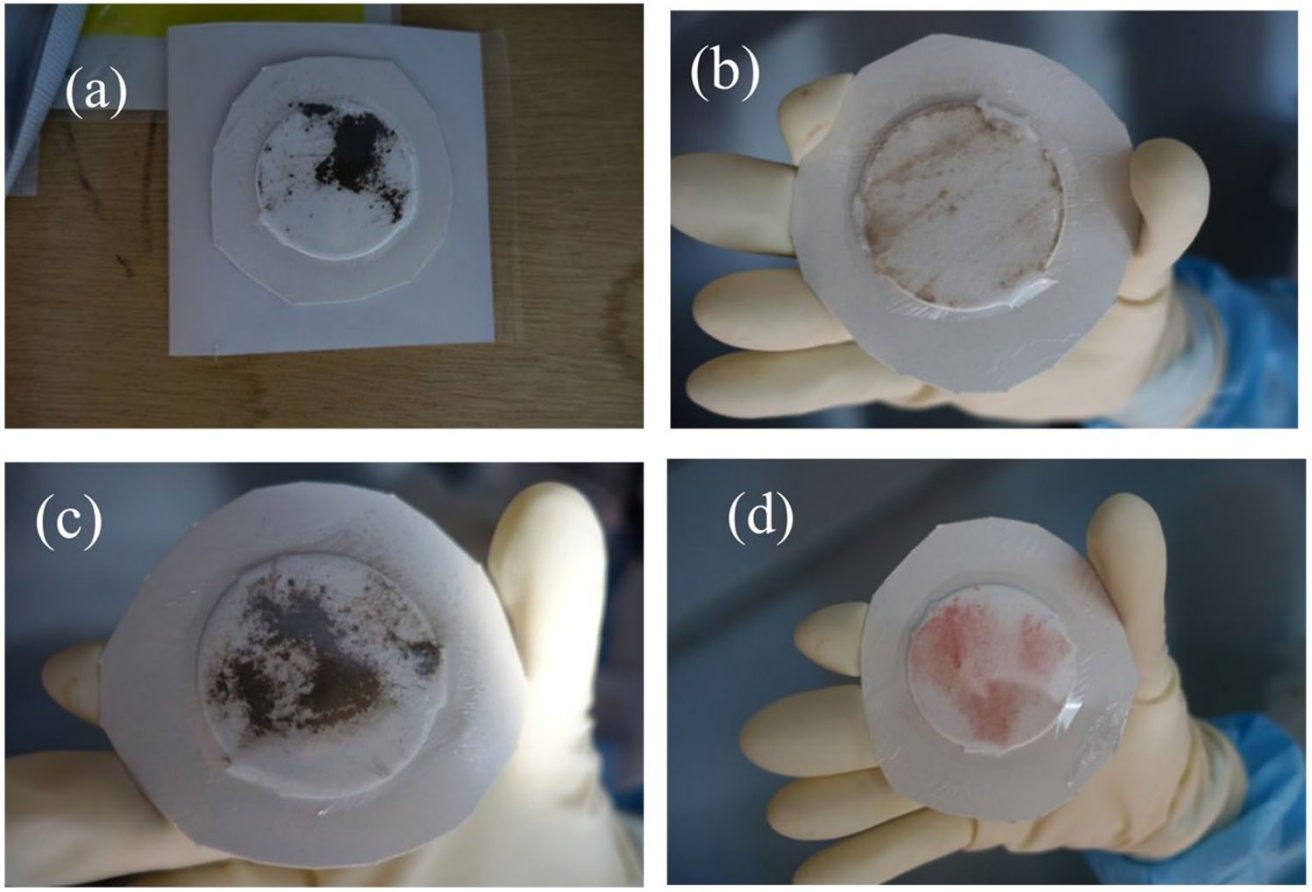
Photographs of smear papers: smear paper nos 1 (**a**), 2 (**b**), 3 (**c**), and 4 (**d**). The photo in no.1 (a) was taken before cutting the outer periphery, but actually, all smear papers were subjected to the same conditions during measurement.

Surface contamination on the floor inside the FDNPP reactor building was wiped off by using smear papers for the measurement. Figure 1 shows photographs of the smear papers. The diameter of the smear papers was 5 cm. The smear papers were covered with a thin polyethylene film to prevent cross-contamination to the detector. The presence of surface dirt on smear papers nos 1, 3, and a little on no. 2, and red paint on smear paper no. 4 was confirmed visually. These smear papers were measured using a ZnS(Ag) survey meter and a Geiger-Muller (GM) counter, and measured results are listed in Table 1. The alpha count rate measured using the ZnS(Ag) survey meter was up to 5,000 cpm. The beta (gamma) count rate measured using the GM counter was higher than the upper measurement limit of the GM counter (>100,000) in the cases of smear papers no. 1 and 4. The beta (gamma) count rate was considerably higher than that of alpha particles (18–1200 times higher). Moreover, it was confirmed that the ZnS(Ag) survey meter was not influenced by beta and gamma rays by comparing the count rates obtained with and without paper.

**Table 1:** List of smear paper numbers, place of wiping, and count rates measured using ZnS(Ag) survey meter, GM counter, and cadmium zinc telluride (CZT) spectrometer.

Smear number	Place of wiping	Count rate [cpm]			Beta/alpha
		ZnS(Ag) survey meter (alpha)	GM counter (beta)	CZT spectrometer (gamma)	
1	Reactor building unit 3	1,100	>100,000	323,434	91
2	Reactor building unit 2	50	60,000	15,410	1200
3	Reactor building unit 3	5,000	90,000	292,960	18
4	Reactor building unit 2	4,100	>100,000	27,542	24

The total number of smear papers was 4. Smear paper no. 3 yielded the highest count rate of 5,000 cpm for alpha particles, whereas smear paper nos1 and 4 yielded the highest count rates of >100,000 cpm for beta particles. Therefore, there was no correlation between the alpha and beta count rates.

### Fundamental performance of the alpha particle imaging detector

**Figure 2:**
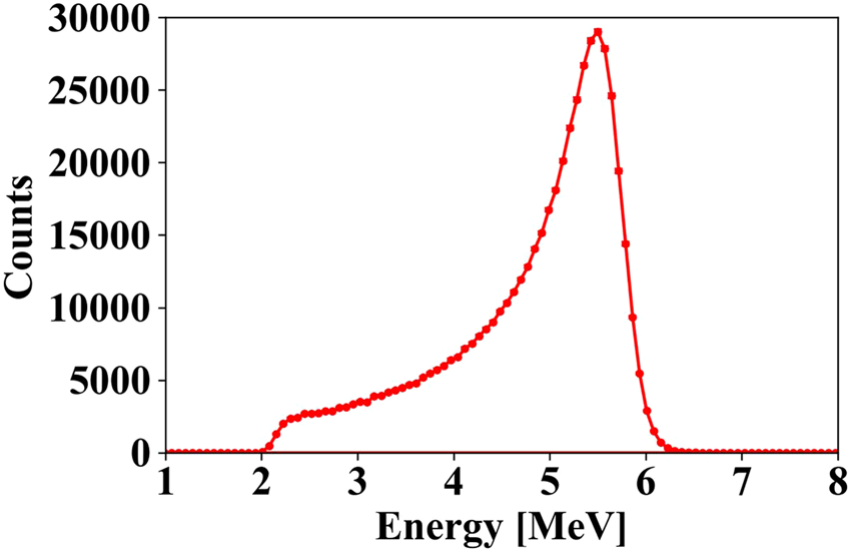
Energy spectrum of 5.5-MeV alpha particles. Error bars indicate standard deviation by taking the square root of the number of counts of each bin in the spectrum.

Figure 2 shows the energy spectrum of 5.5-MeV alpha particles from the ^241^Am source. The energy resolution was calculated by Gaussian fitting. The energy resolution was 13.3% FWHM.

**Figure 3:**
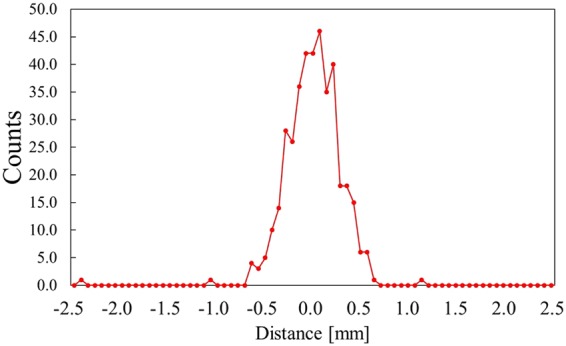
Intensity profile of Pu point source.

The spatial resolution of the developed detector was evaluated by measuring FWHM of an intensity profile of the Pu point source shown in Fig. 3. The spatial resolution was found to be 0.59 mm FWHM.

The count rate of the ^241^Am source measured using the alpha particle imaging detector for 2 min was 132700 cpm (2212 cps). The surface emission rate of the ^241^Am source was 2217 s^−1^. The efficiency was calculated using the following formula^28^:

1 $$\mathrm{Efficiency}[ \% ]=\frac{{\rm{The}}\,{\rm{measured}}\,{\rm{count}}-{\rm{rate}}({\rm{cps}})}{{\rm{The}}\,{\rm{surface}}\,{\rm{emission}}\,{\rm{rate}}\,{\rm{of}}\,{\rm{the}}\,241{\rm{Am}}\,{\rm{source}}({s}^{-1})}\times 100$$

The efficiency was 99.8%. This efficiency was used to calculate surface contamination level in units of Bq/cm^2^.

### Energy spectrum of the FDNPP smear papers measured using the alpha particle imaging detector

**Figure 4:**
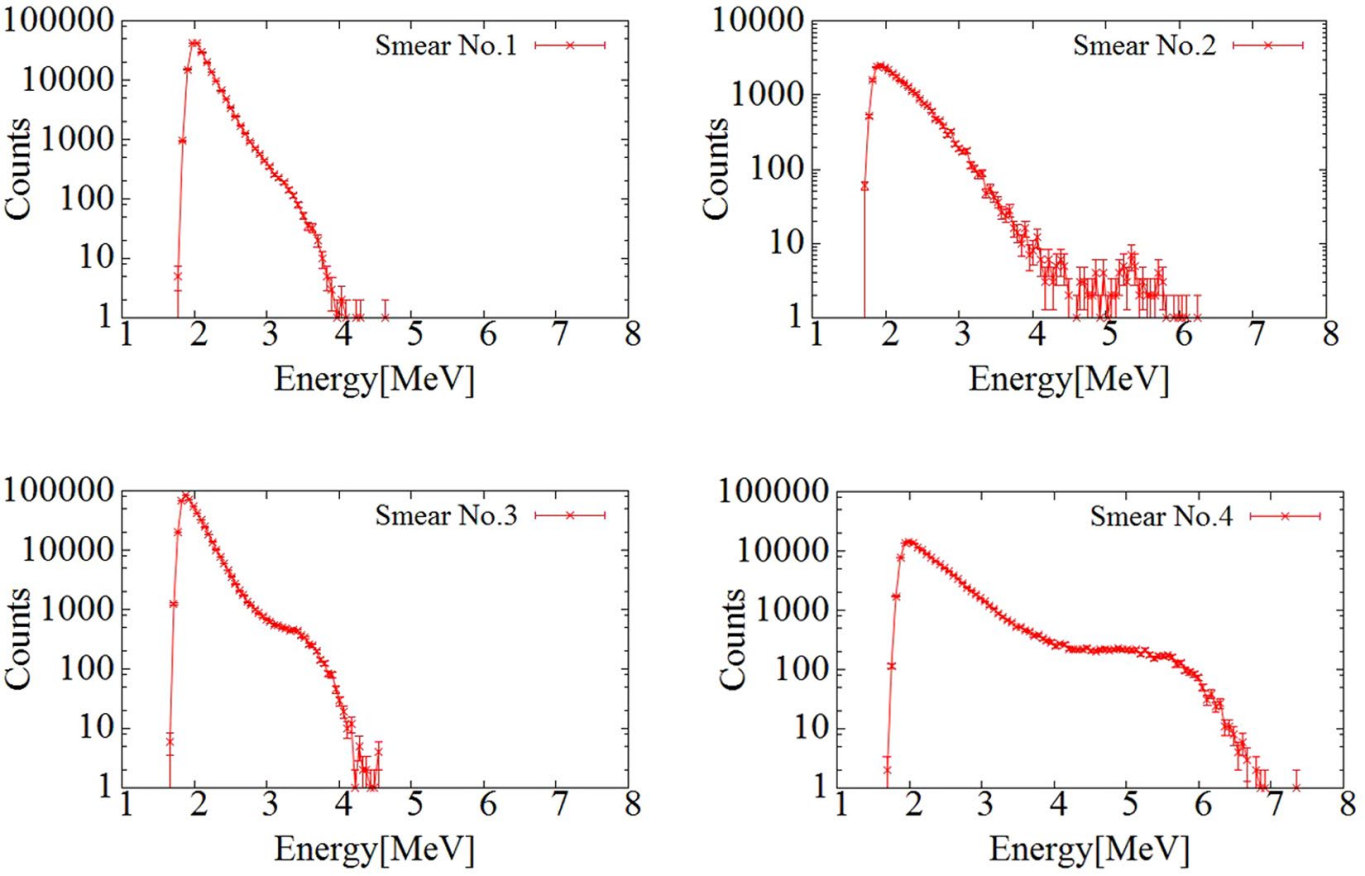
Energy spectra of smear papers nos 1, 2, 3, and 4. Error bars indicate standard deviation by taking the square root of the number of counts of each bin in the spectrum.

Figure 4 shows the energy spectra of the smear papers. In all the measured energy spectra, the peak at 2 MeV was created by beta particles from ^137^Cs and ^90^Sr(^90^Y). However, the shapes of energy spectra of each of the smear papers in the 4–6 MeV region, which corresponds to energies of alpha particles from Pu isotopes (5.5 MeV of ^238^Pu and 5.15 MeV of ^239^Pu and ^240^Pu), were different. In the cases of smear papers no. 1 and 3, the energy spectra were confirmed up to 4 MeV. In the cases of smear papers no. 2 and 4, the energy spectra were confirmed up to 6.5 MeV.

**Figure 5:**
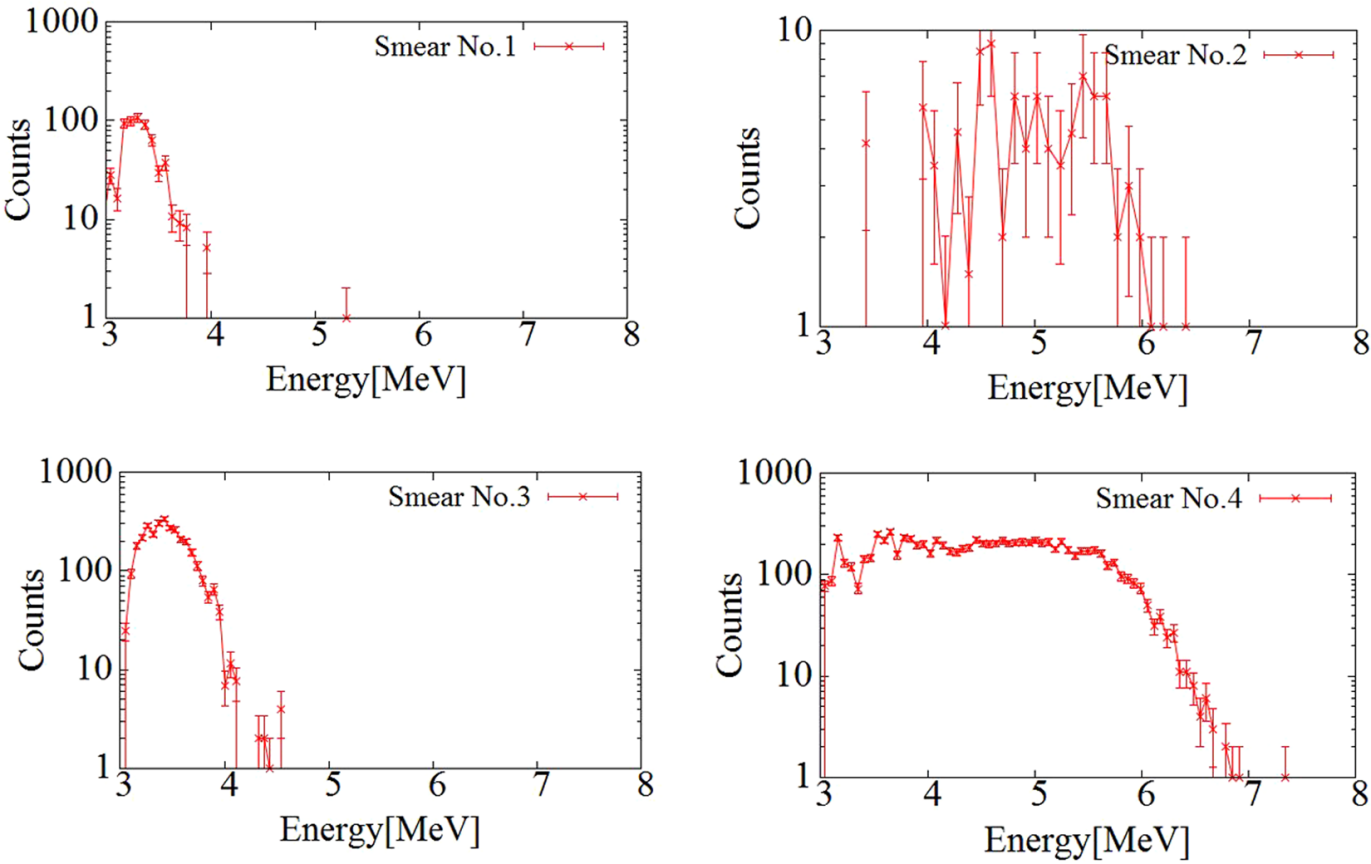
Alpha spectra of smear papers nos 1, 2, 3, and 4. Error bars indicate standard deviation by taking the square root of the number of counts of each bin in the spectrum.

By inserting a paper between the detector and the smear paper, only beta (gamma) spectrum can be measured because alpha particle can be shielded. The alpha spectrum was obtained by subtracting normalized beta spectrum. Figure 5 shows the alpha spectrum of the smear paper. In the cases of smear papers no. 1 and 3, the alpha energy spectrum was confirmed from 3 MeV to 4 MeV. By contrast, in the cases of smear papers no. 2 and 4, the energy spectrum was confirmed from 5 MeV to 6 MeV, which corresponds to an alpha particle energy of 5.5 MeV from ^238^Pu. Dirt particles, for example, soil, can be seen in the photographs of smear papers no.1 and 3. Alpha particles can be absorbed by dirt. and as a result, their energy decreases in cases of smear papers no. 1 and 3. By contrast, smear papers no. 2 and 4 did not contain as many dirt particles as did smear papers no. 1 and 3, and the energies of the alpha particles on those papers were not affected by self-absorption. Therefore, their alpha spectra could be confirmed in the energy range of 5 MeV to 6 MeV.

**Figure 6:**
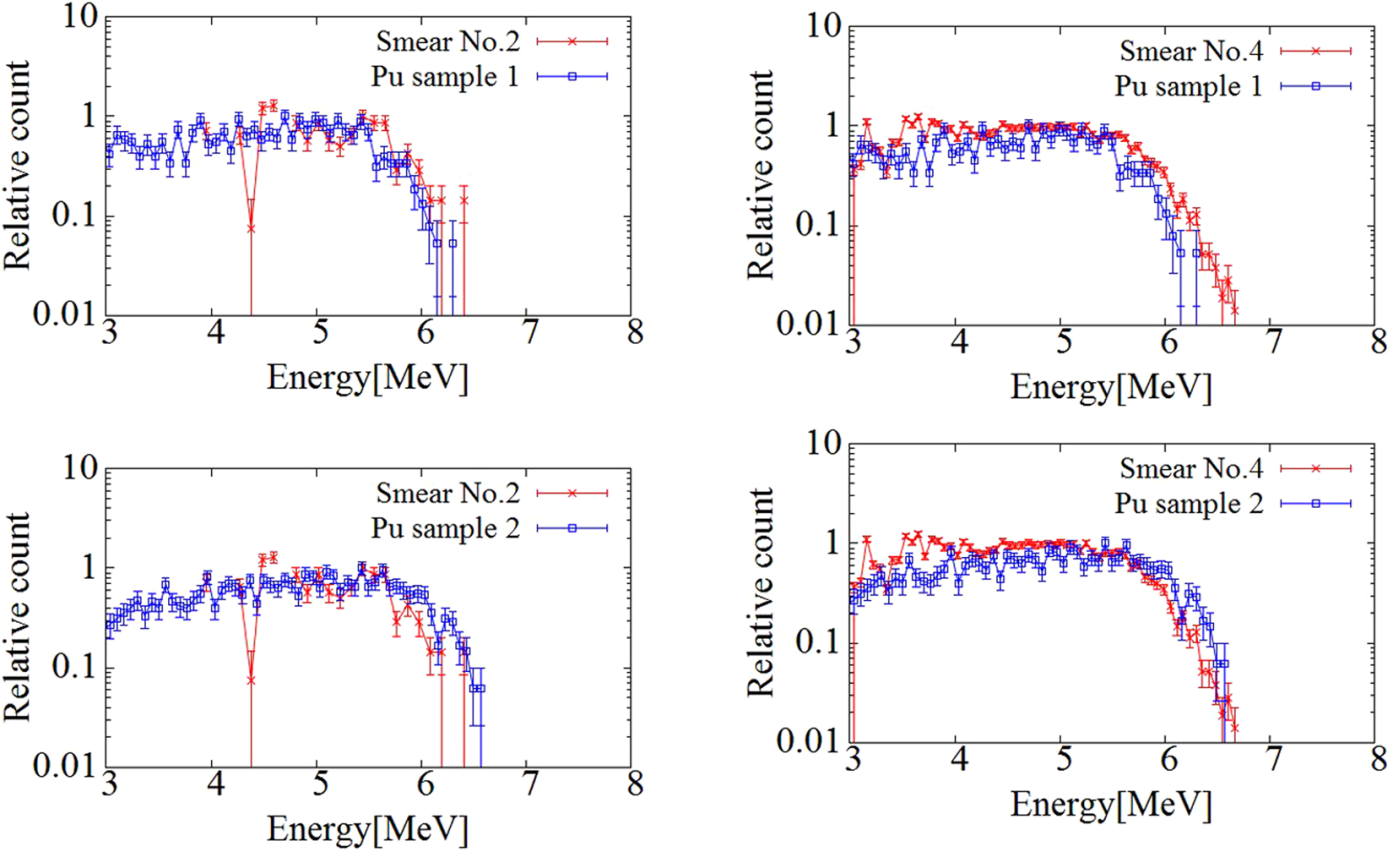
Comparisons of alpha spectra between smear papers and Pu samples measured at nuclear fuel facility. The error bars indicate the standard deviation by taking the square root of the number of counts of each bin in the spectrum.

**Figure 7:**
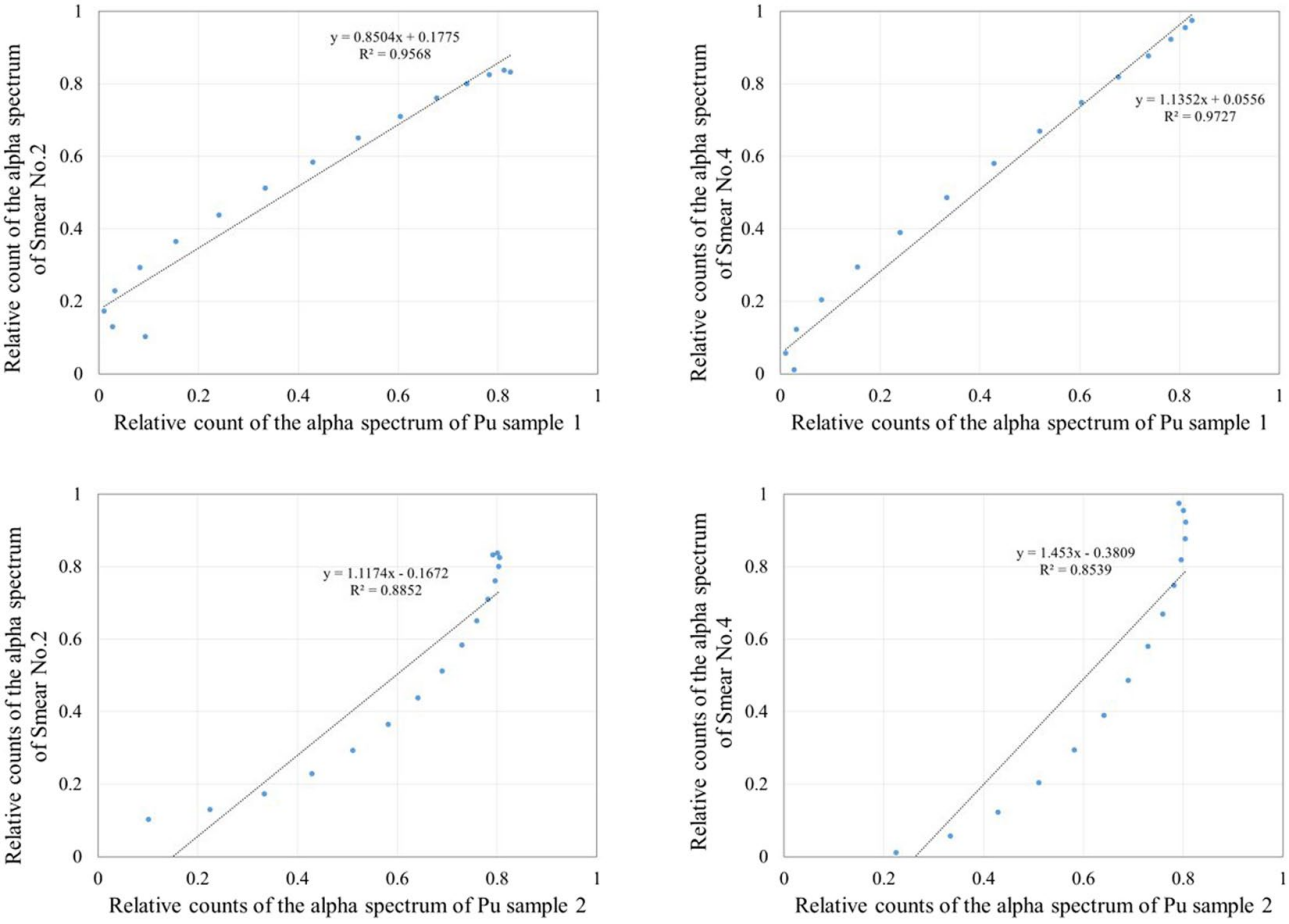
Correlation coefficient of relative counts of alpha spectra of Pu sample and smear papers in energy range of 5–6.5 MeV.

Figure 6 shows comparisons of the energy spectra of the smear papers and the Pu sample measured at the nuclear fuel facility^20^. The energy spectrum of smear papers no. 2 or 4 and that of the Pu sample confirmed up to 6.5 MeV. The shape of the spectra of smear papers no. 2 or 4 and the Pu samples were different because both smear paper and the Pu sample were affected by self-absorption and their peaks were shifted to lower energy. In the results of all smear papers, the alpha spectrum was not confirmed at energies exceeding 6 MeV, which means that this spectrum was not created by naturally occurring radionuclides such as Radon progeny (mainly 7.7 MeV alpha particle from ^214^Po). To show the relationship between the relative counts of the Pu samples and the smear papers quantitatively, the correlation coefficient of the relative counts of the Pu sample and the smear papers in the energy range of 5–6.5 MeV was evaluated. To calculate the correlation coefficient, first, all spectra were fit using polynomial functions to standardize a width of an energy bin in the energy spectrum. Then, the correlation coefficient between the Pu sample and the smear papers was calculated. Figure 7 shows the correlation coefficient of the relative counts of the Pu sample and the smear papers in the energy range of 5–6.5 MeV. The correlation coefficient r was more than 0.93 in all cases, and the relative counts of the Pu sample and the smear papers share a strong, positive linear relationship. Therefore, this alpha spectrum must have been created by artificial radioactivity originating from a nuclear fuel such as ^238^Pu (5.5 MeV alpha particle), ^239^Pu (5.15 MeV alpha particle), and ^241^Am (5.5 MeV alpha particle).

### Two-dimensional distribution of the FDNPP smear papers measured using the alpha particle imaging detector

**Figure 8:**
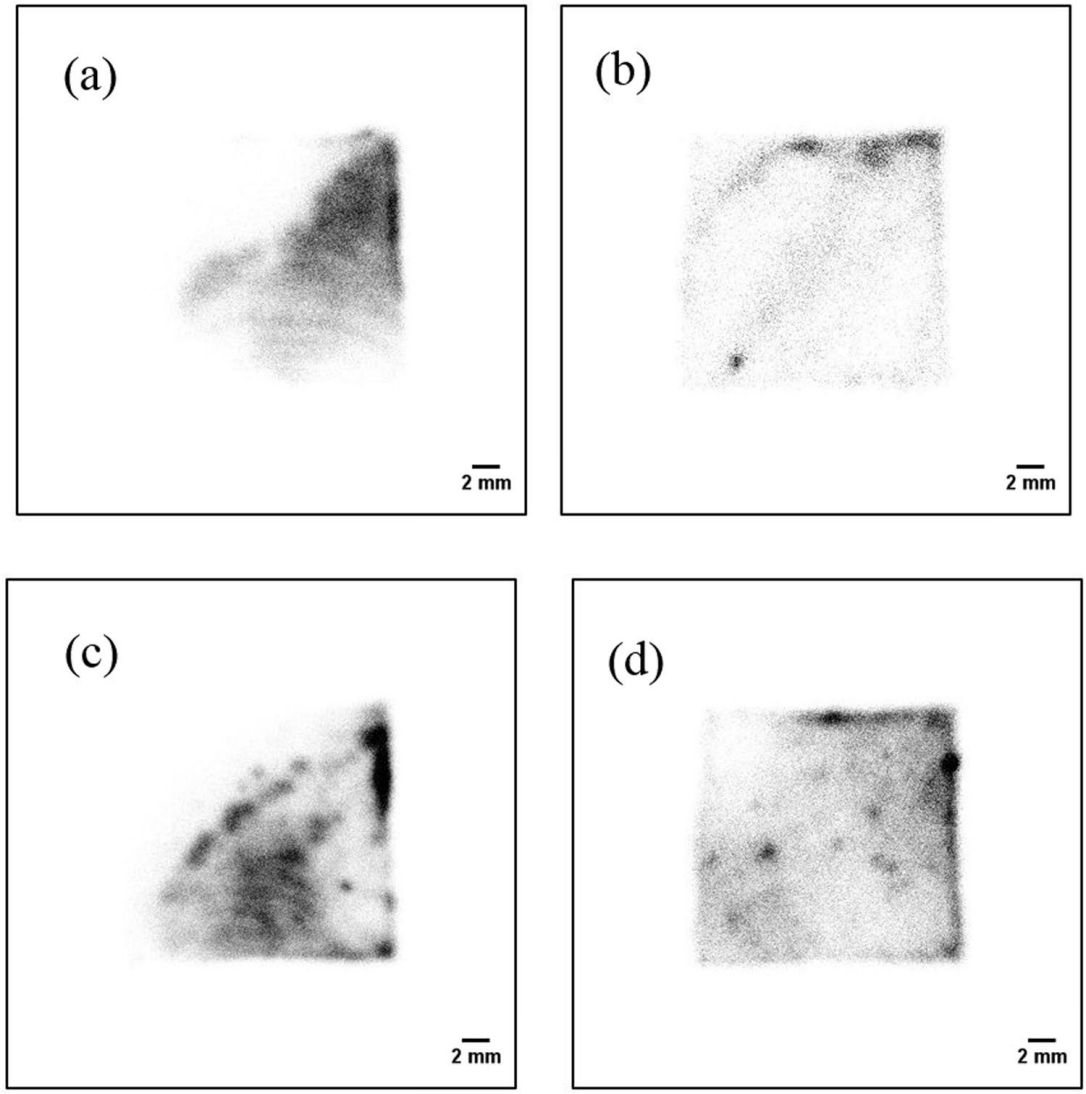
Two-dimensional distributions of the alpha particles on smear papers in energy range of 0–8 MeV (alpha + beta particles): smear paper no. 1 (**a**), 2 (**b**), 3 (**c**), and 4 (**d**).

Figure 8 shows the two-dimensional distribution of the smear papers in the energy range of 0–8 MeV (alpha + beta particles). The two-dimensional distributions of each of the smear papers were different. In the case of smear paper no.1, a pattern with a few streaks was seen. In the case of smear paper no. 2, oblique lines and a spot were seen. In the case of smear paper no. 3, many spots and a patchy pattern were seen. In the case of smear paper no. 4, many spots were distributed in the image.

**Figure 9:**
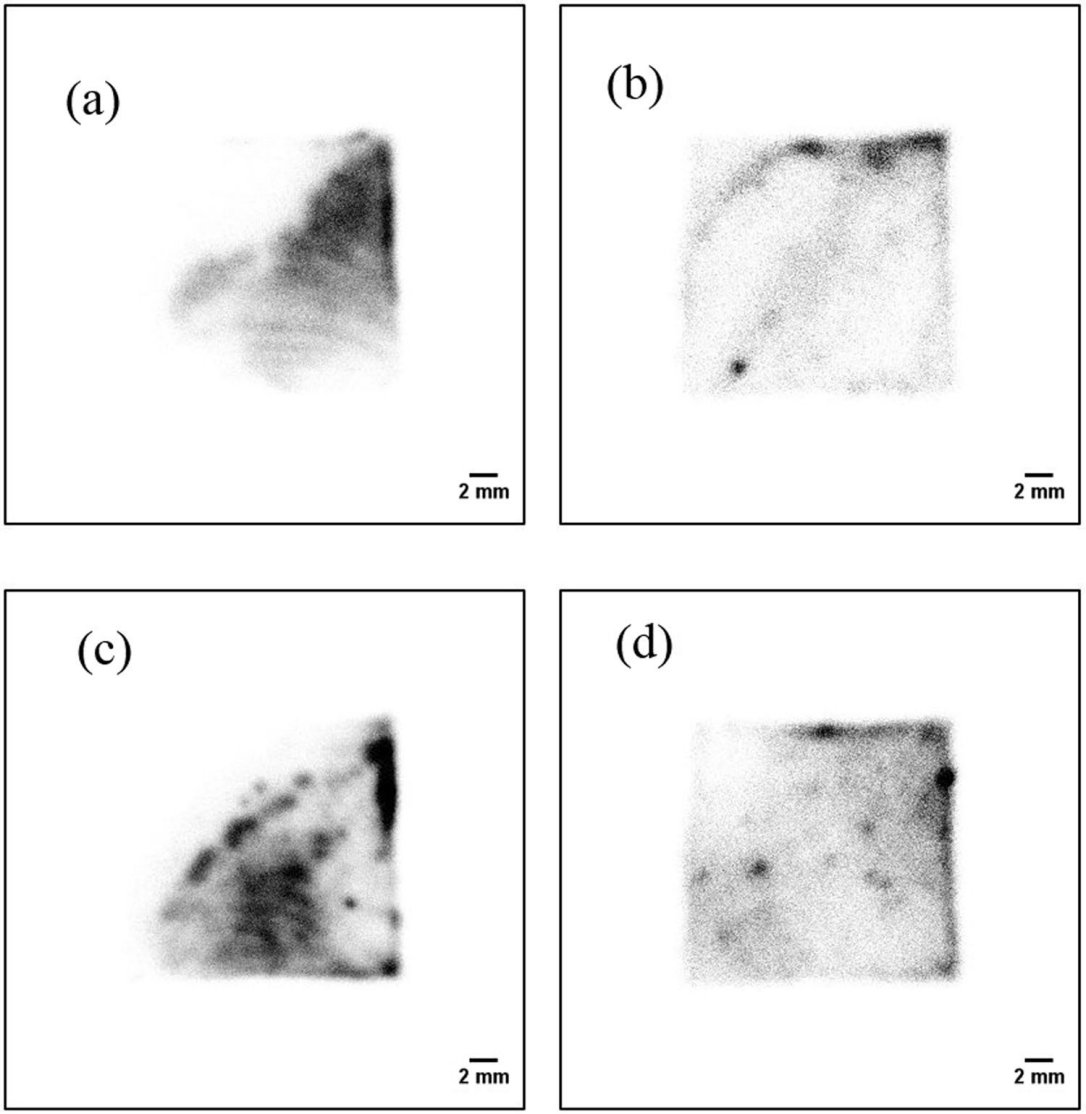
Two-dimensional distributions of smear papers in the energy range of 0–3 MeV (beta particles): smear paper no. 1 (**a**), 2 (**b**), 3 (**c**), and 4 (**d**).

Figure 9 shows the two-dimensional distributions of the smear papers in the energy range of 0–3 MeV (beta particles). The two-dimensional distributions in the 0–8 MeV region and the 0–3 MeV region were similar. Therefore, almost all spots on the two-dimensional distributions were formed by beta particles. It is possible that these particles have been discovered recently in several studies^29,30^.

**Figure 10:**
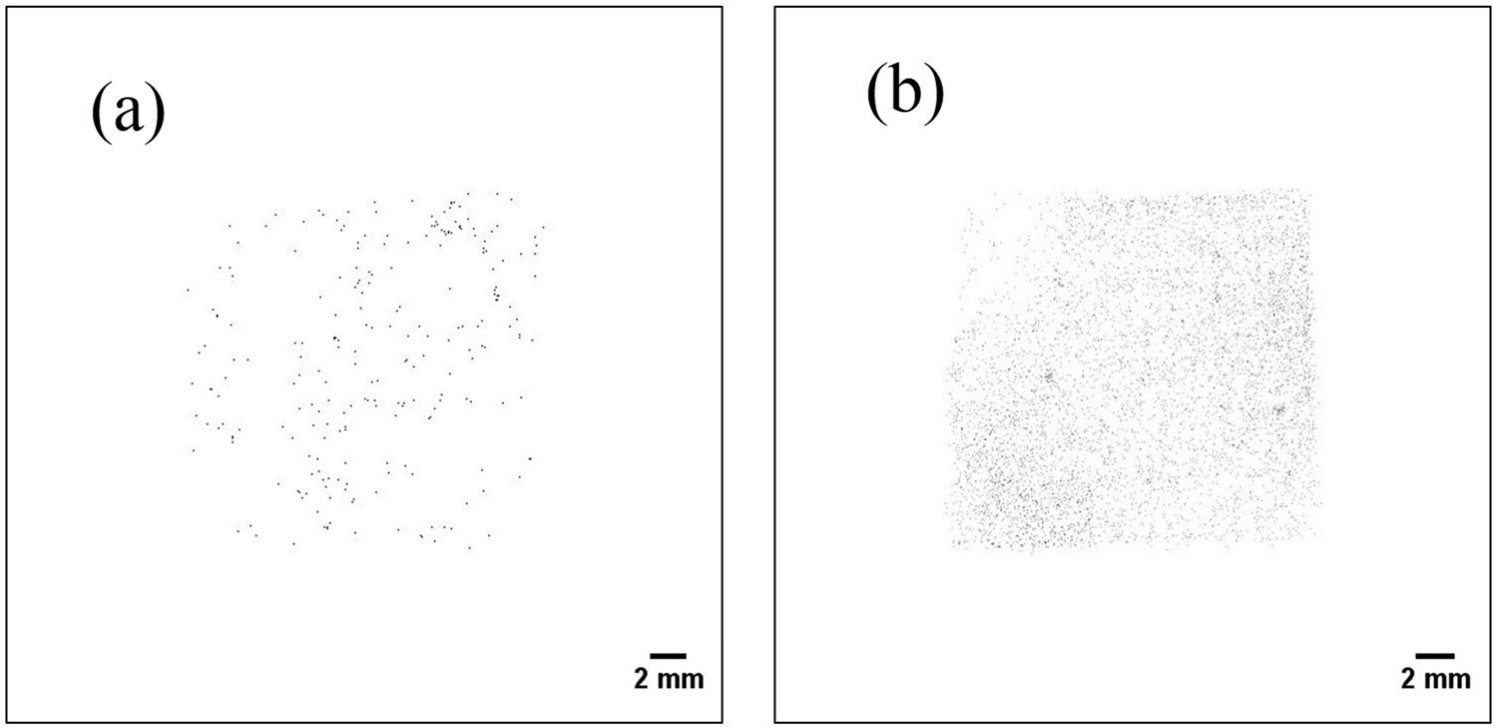
Two-dimensional distribution of the smear papers in energy range of 5–6.5 MeV (alpha particles): smear paper no. 2 (**a**) and 4 (**b**).

**Figure 11:**
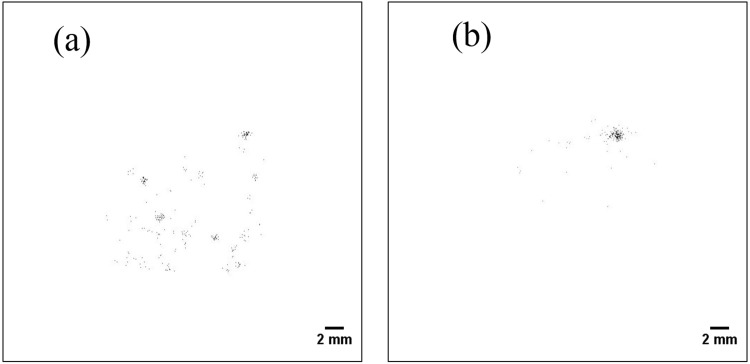
Two-dimensional distribution of Pu samples measured at nuclear fuel facility in energy range of 5–6.5 MeV: Pu sample 1 (**a**), Pu sample 2 (**b**).

**Figure 12:**
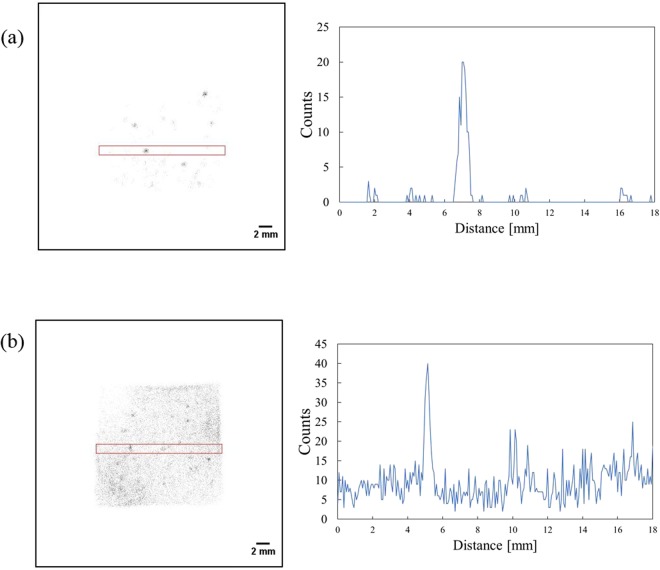
Comparison of two-dimensional distribution measured in 30 min in the energy range of 5–6.5 MeV(Left) and intensity profile(Right): Pu sample 1 (**a**), smear paper no. 4 (**b**). The red-square indicates a region of interest to measure the intensity profile.

Figure 10 shows the two-dimensional distribution of the smear papers in the energy range of 5–6.5 MeV (alpha particles). Alpha particle emitters were widely distributed on the smear paper, as opposed to forming spots. In addition, Fig. 11 shows the two-dimensional distribution of the Pu sample obtained from a fabrication process of mixed oxide (MOX) fuel in the nuclear fuel facility. These two-dimensional distribution images were created in the same energy range of 5–6.5 MeV. The spots were confirmed in both images of the Pu samples. Even though the energy ranges of the FDNPP smear paper and the Pu sample are identical (5–6.5 MeV), their two-dimensional distributions are different. To confirm this two-dimensional distribution of the smear paper no.4 in detail, which is possible that Pu isotope exists, was measured for 30 min. Figure 12 shows comparisons of the two-dimensional distribution and intensity profile measured in 30 min in the energy range of 5–6.5 MeV: Pu sample 1 (a), smear paper no. 4 (b). In the intensity profile of Pu sample 1, the maximum count of 20 can be confirmed at the distance of 7.0 mm. In contrast, in the intensity profile of smear paper no. 4, the maximum count of 40 can be confirmed at the distance of 5.1 mm, whereas counts of other area fluctuated between 4 to 25. It is possible that minute particles and large particles were mixed in one smear paper.

### Gamma-ray spectrum of the FDNPP smear papers using a CZT-based gamma spectrometer

**Figure 13:**
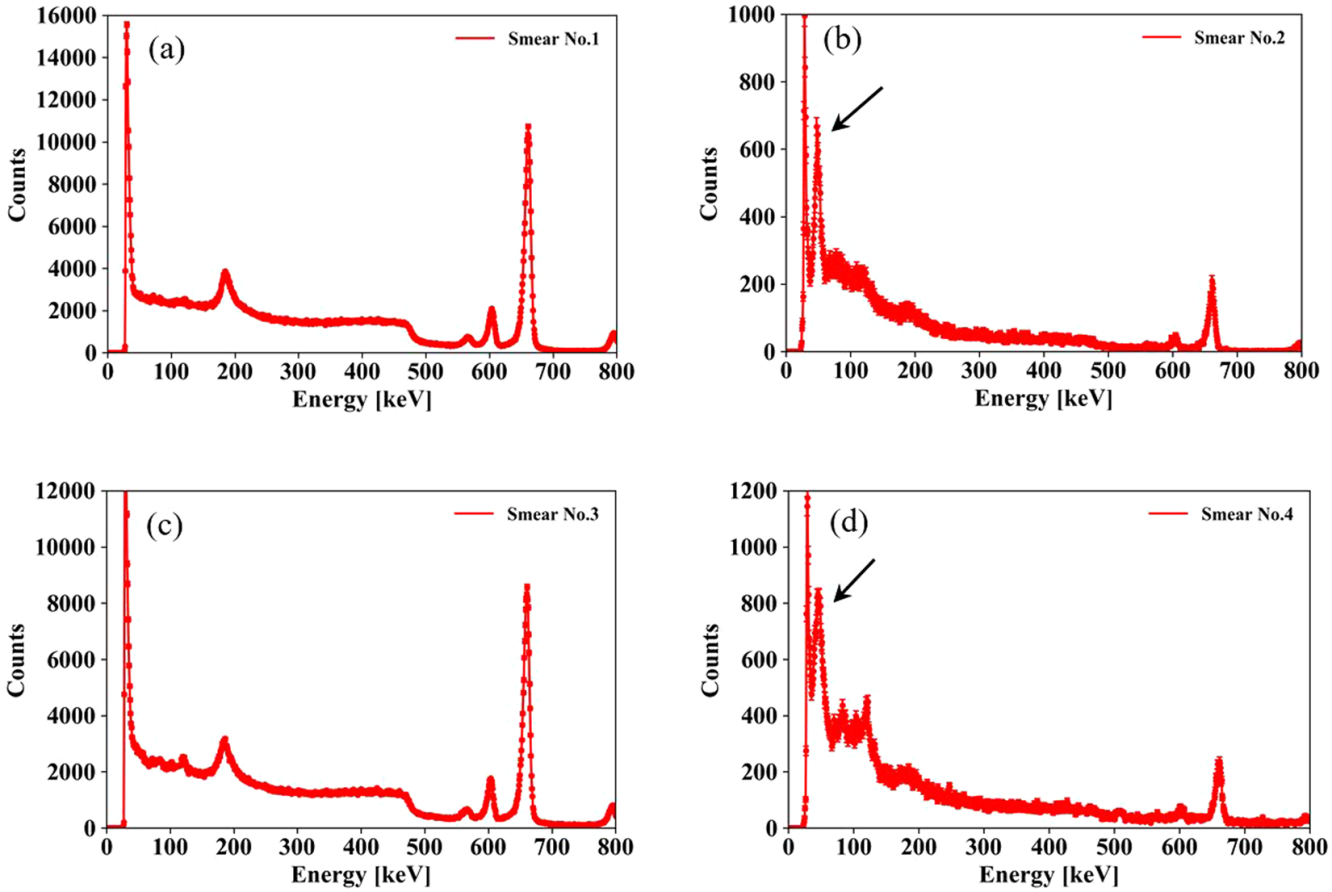
Gamma-ray spectra of smear papers measured using CZT-based gamma spectrometer: smear papers no. 1 (**a**), 2 (**b**), 3 (**c**), and 4 (**d**). Error bars indicate standard deviation by taking the square root of the number of counts of each bin in the spectrum. The arrows indicate the ^241^Am peak.

Figure 13 shows the gamma-ray spectra of the smear papers measured using a CZT-based gamma spectrometer. In the spectra of smear paper no. 2 and 4, a peak at 60 keV was confirmed, and it was formed by gamma rays from ^241^Am. In the spectra of smear papers no. 1 and 3, the peak at 60 keV was not confirmed because the count rates of ^134^Cs and ^137^Cs were too high and the peak was buried in the Compton continuum.

### Surface contamination level of smear paper

The measured alpha count was converted to surface contamination (SC) in Bq/cm^2^ and it was compared with that obtained using the ZnS(Ag) survey meter. The SC can be calculated using the following formula^31^:

2 $${\rm{SC}}(Bq/c{m}^{2})=\frac{Measured\,count[cps]}{S\times F\times {\varepsilon }_{s}\times {\varepsilon }_{i}}\times {\rm{area}}\,{\rm{ratio}}$$

where S represents wiped area [cm^2^] (=100 cm^2^), F represents removal factor (=0.1, as recommended in ISO-7503-1^32^), ε_s_ denotes source efficiency (=0.25), and ε_i_ denotes instrument efficiency. The instrument efficiency of the alpha particle imaging detector was 99.8% and that of the ZnS(Ag) survey meter was 32.5%/2π ± 25%, which is more than 30%/2π^33^. Because the effective area of the alpha particle imaging detector was smaller than the area of the smear paper (5 cm diameter), area ratio (=area of smear paper/ effective area of alpha particle imaging detector) was applied as a calibration factor.

**Figure 14:**
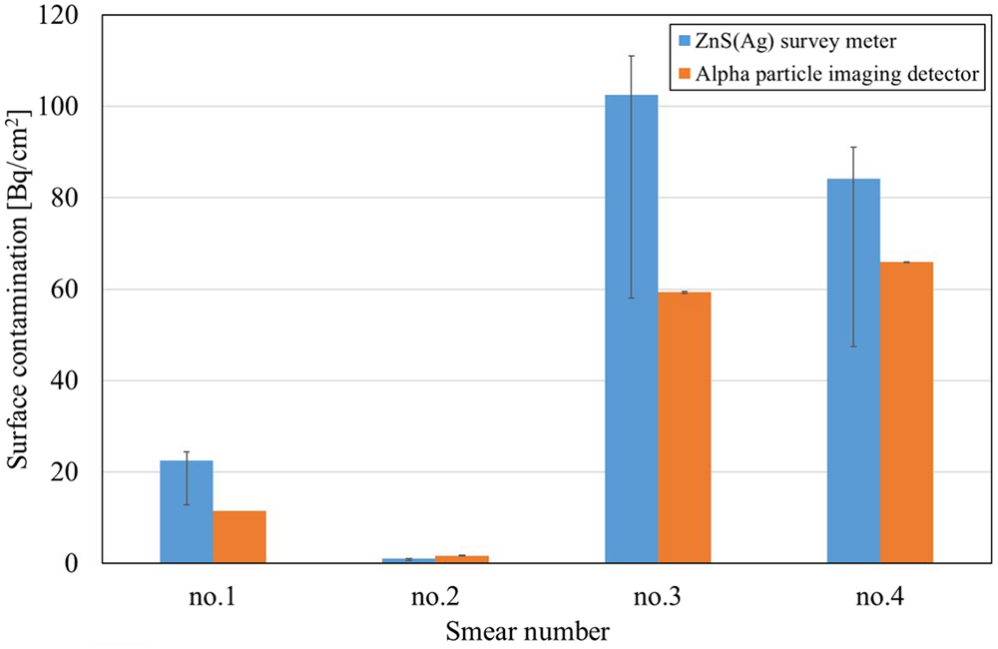
Comparison of surface contamination measured using ZnS(Ag) survey meter and alpha particle imaging detector. The ZnS(Ag) survey meter deviation of ±25% and the standard deviation of the two measurements of the alpha particle imaging detector are shown as error bars.

Figure 14 shows the SC level of the smear papers. Because the ZnS(Ag) survey meter has a wide range of instrument efficiency, the SC levels measured by the ZnS(Ag) survey meter were 12.8–24.4 Bq/cm^2^ (smear paper no. 1), 0.6–1.1 Bq/cm^2^ (no. 2), 58.0–111.1 Bq/cm^2^ (no. 3), and 47.5–91.1 Bq/cm^2^ (no. 4). By contrast, the SC levels measured by the alpha particle imaging detector were 11.5 Bq/cm^2^ (smear paper no. 1), 1.7 ± 0.06 Bq/cm^2^ (no. 2), 59.3 ± 0.21 Bq/cm^2^ (no. 3), and 65.9 ± 0.03 Bq/cm^2^ (no.4). The standard deviation of repeat measurements was minimal. The SC measured using the ZnS(Ag) survey meter corresponded to the alpha particle imaging detector within the variation of instrument efficiency of the ZnS(Ag) survey meter.

### Evaluation of Air contamination level

We evaluated air contamination from the measured SC level using following formula.

3 $${\rm{The}}\,{\rm{resuspension}}\,{\rm{factor}}\,{\rm{K}}\,({{\rm{cm}}}^{-1})=\frac{Air\,contamination\,[Bq/c{m}^{3}]}{Surface\,contamination\,[Bq/c{m}^{2}]}$$

where, the value of K for plutonium is 2 × 10^−8^ cm^−1^ ^31^. The maximum SC level measured by the alpha particle imaging detector was 65.9 ± 0.03 Bq/cm^2^ (smear no.4). The air contamination can be calculated to be 1.3E-6 Bq/cm^3^.

## Discussion

We measured the existing alpha emitter contamination in smear papers at the FDNPP site by using the alpha particle imaging detector, and revealed that the alpha emitters in the reactor buildings at the FDNPP site originated from the nuclear fuels. This result has been impossible to obtain using ZnS(Ag) detectors. The correlation coefficient of the alpha spectra of the smear papers and the Pu sample had a strong positive linear relation. The Pu sample was obtained from the MOX fuel facility. Although ^239^Pu is the dominant Pu isotope in the MOX fuel (^238^Pu: 3.5%, ^239^Pu: 47.4%)^34^, ^238^Pu has the highest specific activity (^238^Pu: 600 GBq/g, ^239^Pu is 2 GBq/g) among the plutonium isotopes. Considering the specific activity and abundance ratio of Pu isotopes, 5.5 MeV alpha particles from ^238^Pu majorly contribute to the alpha spectra. The form of Pu on the smear paper will be PuO_2_ if it originated from the fuel^35^. Moreover, the ^241^Am peak at 60 keV was found by measuring the gamma spectrum. ^241^Am is a decay product of ^241^Pu and is often measured as the evidence of Pu contamination. Furthermore, in an evaluation report of the amount of radioactive nuclides in the reactor core by using the ORIGEN2 code, ^238^Pu and ^241^Am that emit 5.5 MeV alpha particles showed high activities among the alpha emitters in nuclear fuel (e.g., in the reactor building unit 2 after 5 years, ^238^Pu:4.87E+6GBq/core, ^239^Pu:8.95E+5GBq/core, ^240^Pu:1.04E+6GBq/core, and ^241^Am:2.43+6GBq/core)^36^. Based on these results, the major alpha emitters on the smear papers are ^238^Pu and ^241^Am.

The beta activity of the FDNPP smear papers was approximately 10–1000 times higher than their alpha activity. Therefore, the influence of beta particles must be considered in alpha spectrum measurement. In this study, we used a very thin (0.05-mm-thick) scintillator to reduce the influence of beta particles. However, in measured spectra of the smear papers, beta spectrum and alpha spectrum overlapped partly. By subtraction with/without paper, the beta spectrum could be removed. In the future, a technique for discrimination of beta particles is necessary for accurate alpha spectrometry.

The two-dimensional distribution of alpha particles on the FDNPP smear papers was different from that of the Pu sample; In the Pu sample, spots were observed, but spots and wide distribution of minute particles were simultaneously observed in the FDNPP smear papers. The Pu sample originated from particles used to fabricate MOX fuel, and its diameter was approximately a few micrometers^12,37–39^. However, the alpha emitters in FDNPP would have different diameters and compositions compared to the Pu particle in the MOX fuel fabrication facility. Further studies are required to clarify the diameter and composition of the Pu in the FDNPP.

Sato *et al*. subjected a rubble sample from reactor unit 2 to radiochemical analysis^40^. The radioactive concentration was (6.1 ± 0.3) × 10^1^ Bq/g of ^238^Pu, (2.5 ± 0.2) × 10^1^ Bq/g of ^239+240^Pu, (2.4 ± 0.2) × 10^1^ Bq/g of ^241^Am, and (5.1 ± 0.3) × 10^1^ Bq/g of ^244^Cm. Our measurement smear paper no. 4 showed that the radioactive concentration of alpha particles was (6.6 ± 0.003) × 10^1^ Bq/cm^2^. Both measurement results are of the same order of magnitude.

Given that the SC levels of three of the four smear papers easily exceeded 4 Bq/cm^2^ which is considered an acceptable level in Japan, the level of contamination can be thought to be extremely high. This contamination is loose as opposed to being fixed because it can be removed using smear papers. It can lead to internal exposure, and therefore, extreme caution is required to prevent inhalation of Pu contamination. A worker entering into FDNPP must put on a full-face mask. Considering the resuspension factor (2 × 10^−8^ cm^−1^) and the protection factor of the full-face mask (generally, 50), the air concentration inside the mask is estimated to be below the air concentration limit of ^238^Pu (7 × 10^−7^ Bq/cm^3^)^41^.

We detected many beta-emitting particles with extremely high activity. The combination of multiple technologies such as Transmission Electron Microscope is useful to identify them^29,30^.

A chemical process is very difficult to apply on-site at the FDNPP. In Sato’s chemical analysis, the samples had to be transported from the FDNPP to the Nuclear Science Research Institute of the Japan Atomic Energy Agency^40^. Even though our measurements were conducted on-site at the FDNPP without destroying the samples, the results showed the radioactive concentration of alpha particles, which was of the same order of magnitude as measured using a radiochemical analysis. Therefore, our measurement method proved to be valuable for detecting alpha emitter contamination at the FDNPP site.

## Methods

### Alpha particle imaging detector

**Figure 15:**
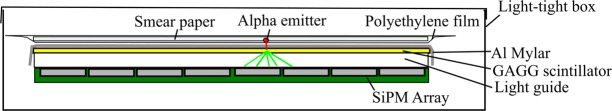
Schematic drawing of alpha particle imaging detector.

**Figure 16:**
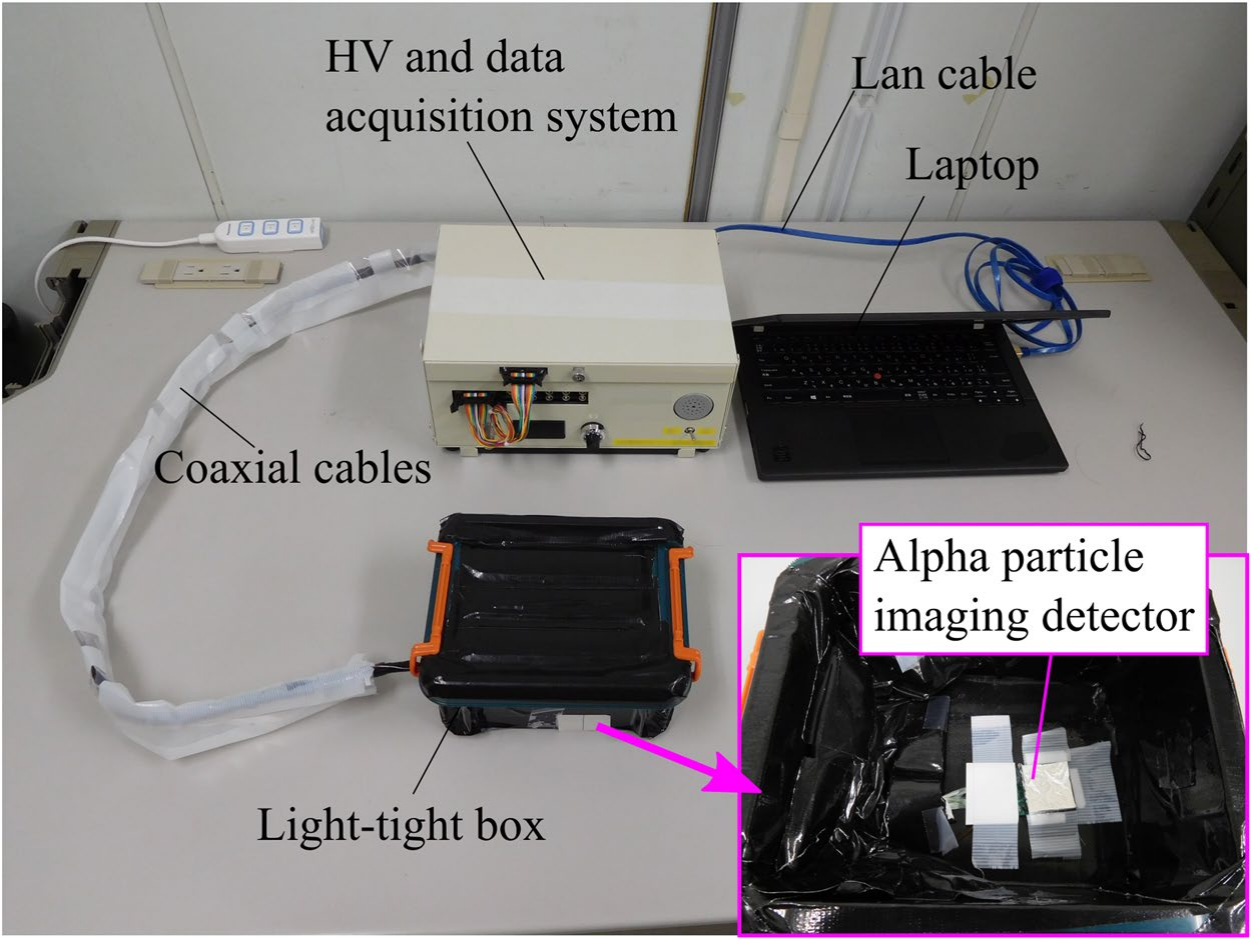
Photograph of alpha particle imaging detector and data acquisition system.

An alpha particle imaging detector must have good energy resolution for identifying radionuclides of alpha emitters. Moreover, because there are large quantities of beta and gamma emitters at the FDNPP site, the influences of beta and gamma rays must be distinguished from those of alpha particles. Figure 15 shows a schematic drawing of the alpha particle imaging detector developed in this study. A very thin (0.05-mm-thick) GAGG scintillator was used employed to limit beta sensitivity^42^. The length and the width of the scintillator size 26 mm × 26 mm. The 0.05-mm-thick GAGG scintillator was coupled to a 1-mm-thick glass plate and a 1-mm-thick acrylic light guide. The bottom of the acrylic light guide was optically coupled to silicon photomultiplier (SiPM) arrays having 8 × 8 channels (Through Silicon Via (TSV) MPPC array S12642-0404PA-50, Hamamatsu Photonics K.K., Japan) by using an optical compound (Shin-Etsu silicone, Shin-Etsu Chemical Co., Ltd., Japan). The detector was covered with aluminized mylar to shield it from external light. The GAGG scintillator produces scintillation light when an alpha particle deposits energy. The SiPMs convert this scintillation light into electric signals. The analog signals produced by the 8 × 8 SiPMs were fed to preamplifiers and a weight-summing circuit, which creates X+, X−, Y+, Y− signals for position calculation. These signals are converted to digital signals by using analog-to-digital converters and subsequently fed to a field programmable gate array. Finally, the data are transferred to a laptop via a lan cable, and the two-dimensional position and energy spectrum of the alpha particles are displayed on the laptop. Figure 16 shows a photograph of the alpha particle imaging detector and the data acquisition system. The data acquisition system is the same as that in the previous alpha imaging system^20^. High voltage (HV) was adjusted to −67 V, and the temperature of the SiPM circuit was monitored to compensate for temperature-dependent changes in gain. During measurement, the detector and sample were enclosed in a light-tight box to shield them from external light.

### Fundamental performance of alpha particle imaging detector

Before the measurement at the FDNPP, we confirmed the fundamental performance, that is, energy resolution, spatial resolution, and efficiency, of the alpha particle imaging detector. Energy resolution and efficiency were evaluated using an ^241^Am alpha source of diameter 5 mm that emitted 5.5 MeV alpha particles. A thin polyethylene film was placed between the source and the alpha imaging detector to create the same conditions as those during the measurement of samples at the FDNPP site. Spatial resolution of the developed detector was evaluated using a Pu point source. We got the predicted standard deviation by taking the square root of the number of counts of each bin in the spectrum^28^.

### Measurement of smear papers by using alpha particle imaging detector

**Figure 17:**
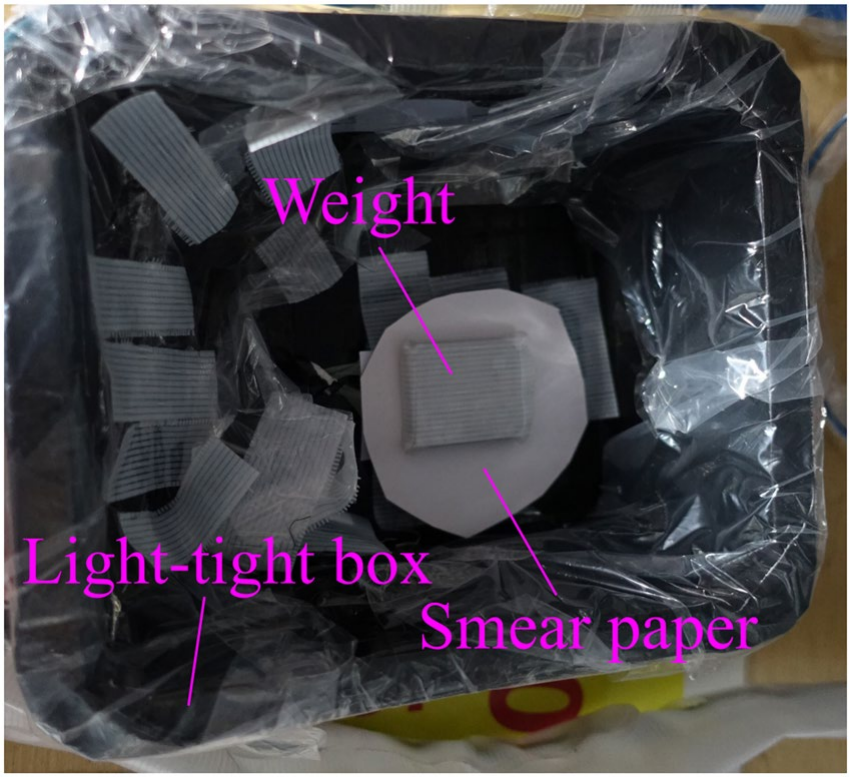
Photo of measurement of smear papers: Top view. The alpha particle imaging detector was placed under the smear paper.

Figure 17 shows a schematic drawing of the measurement of the smear papers by using the developed alpha particle imaging detector. The alpha particle imaging detector and the smear papers were placed in a container in the field of the FDNPP. Each of the smear papers was placed on the detector and close contact between the paper and the detector was achieved by placing a weight on top. The smear paper was placed in a light-tight box, and the door of the container was closed to prevent penetration of external light. The measurement was repeated twice during a 5 min period. Also, the measurement was started at around noon to maintain the room temperature. The dose-rate of gamma rays in the measurement environment was approximately 10 μSv/h. Moreover, we measured two Pu samples obtained from a MOX fuel fabrication facility for comparison with the FDNPP smear papers. The measurement was conducted under the same conditions as those used in FDNPP (measurement time, HV).

### Measurement of smear papers by using a cadmium zinc telluride (CZT)-based gamma spectrometer

The smear papers were also measured using a CZT-based gamma spectrometer (GR1-Spectro Gamma Ray Spectrometer, Kromek Group plc, United Kingdom). This measurement aimed to identify the 60-keV peak of ^241^Am. Each sample was measured for 5 min.
